# Neurodegeneration in an adolescent with Sjogren-Larsson syndrome: a decade-long follow-up case report

**DOI:** 10.1186/s12881-018-0663-0

**Published:** 2018-08-29

**Authors:** Kye Hee Cho, Sung Han Shim, Youngsoo Jung, Se Ra Sung, MinYoung Kim

**Affiliations:** 1 Department of Rehabilitation Medicine, CHA Bundang Medical Center, CHA University, 59 Yatap-ro, Bundang-gu, Seongnam, Gyeonggi-do 13496 Republic of Korea; 2 Department of Biomedical Science, College of Life Science, CHA University, 120 Haeryong-ro, Pochun, Gyeonggi-do 11160 Republic of Korea; 3 Genetics Laboratory, Fertility Center, CHA Gangnam Medical Center, CHA University, Seoul, 06135 Republic of Korea

**Keywords:** Neurodegeneration, Sjogren-Larsson syndrome, Dystonia, Neurologic deterioration

## Abstract

**Background:**

Sjogren-Larsson syndrome is a hereditary neurocutaneous syndrome that is non-progressive in nature. Although neuroregression has been reported in seizure-prone preschool children requiring anti-epileptic treatment, teenage-onset dystonia precipitating neurodegeneration without any immediate causal events has yet to be reported.

**Case presentation:**

We describe a young woman with spastic diplegia and intellectual disability who began to show progressive neurological deterioration from 12 years of age, with the onset of dystonia and tremor. She was initially diagnosed with spastic cerebral palsy and periventricular leukomalacia based on brain magnetic resonance imaging. Follow-up brain imaging from 13 years of age did not reveal apparent changes, though abnormal electroencephalographic findings occurred in parallel with her decline in motor function. By 19 years of age, she had developed dysphagia and became completely dependent on others for most activities of daily living. Ultimately, whole-exome sequencing revealed a heterozygous compound mutation in the *ALDH3A2* gene that corresponds to Sjogren-Larsson syndrome: an exon 9 deletion (1291-1292delAA) from the mother and an exon 5 splicing mutation (798 + 1delG) from the father. Neuroregression has been reported in preschool children after seizures requiring treatment, though our patient did not experience any immediate causal events. This report summarizes the clinical, radiologic, and electrophysiological findings observed over a decade concurrent with neurological deterioration after the onset of dystonia and tremor at the age of developmental ceiling in Sjogren-Larsson syndrome.

**Conclusions:**

In addition to the influence of additive variants or other environmental factors, accumulation of metabolites due to defective fatty aldehyde dehydrogenase is a potential pathomechanism of neurodegeneration in this patient. Neurological deterioration may be a presentation that is unnoticed in Sjogren-Larsson syndrome due to the rarity of the disease. This report highlights a unique clinical feature of Sjogren-Larsson syndrome with progressive neurodegeneration associated with dystonia and tremor.

**Electronic supplementary material:**

The online version of this article (10.1186/s12881-018-0663-0) contains supplementary material, which is available to authorized users.

## Background

Sjogren-Larsson syndrome (SLS) is an autosomal recessive disorder (OMIM#270200) characterized by spastic di−/quadriplegia, intellectual disability, and generalized ichthyosis [1, 2]. In general, cerebral palsy is the most common cause of motor disability in childhood that usually accompanies limb spasticity [3], and both disorders share similarities of spasticity and a non-progressive nature in their clinical course. An SLS patient initially diagnosed with spastic cerebral palsy as a toddler began to show progressive deterioration in adolescence during over-decade-long follow-up. Dystonia and tremor developed at 12 years of age and precipitated neurological deterioration; she was eventually diagnosed with SLS based on the results of whole-exome sequencing. This report describes progressive neurodegeneration associated with dystonia and tremor in an SLS patient. The results of serial brain imaging, electroencephalography, and evoked potential studies are discussed with the results of a literature review regarding the possible pathomechanisms of neurodegeneration.

## Case presentation

**Fig. 1:**
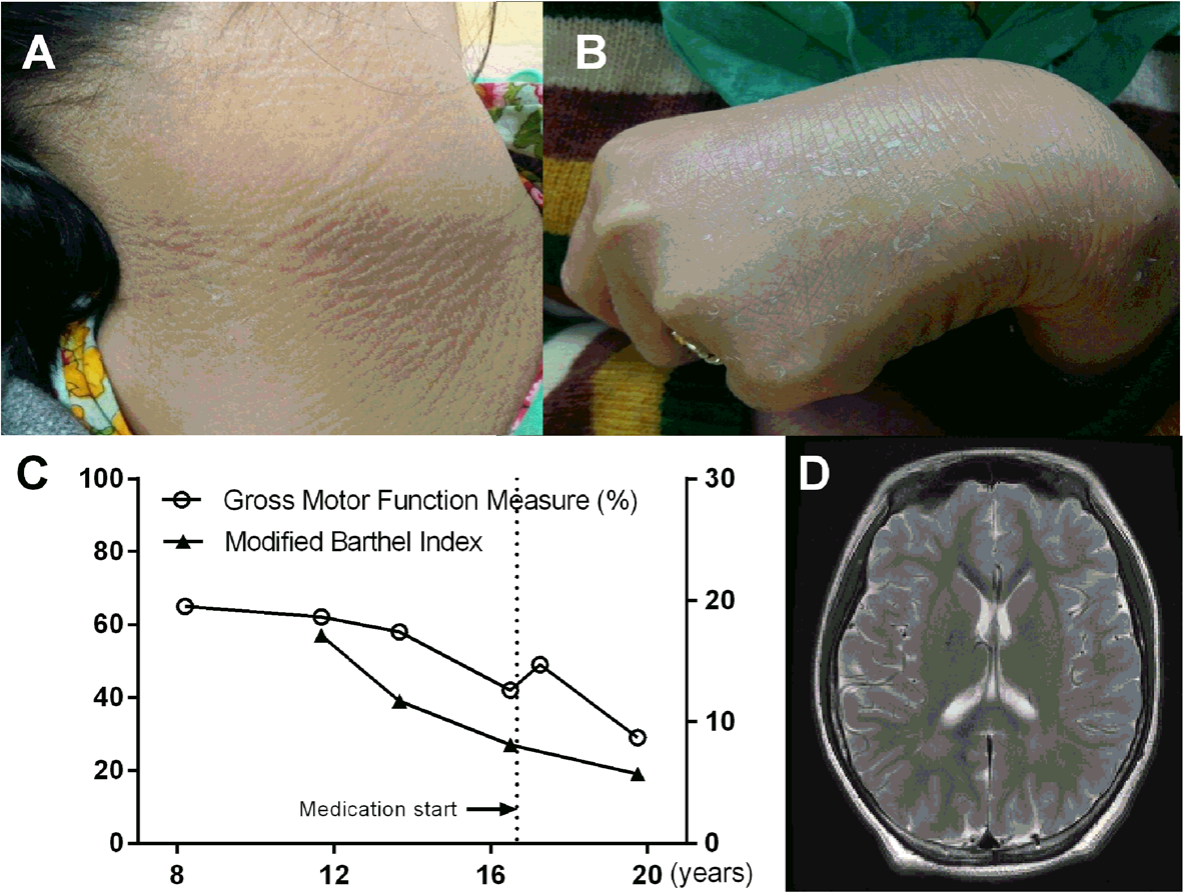
Photographs of ichthyosis on the (**a**) posterior neck and (**b**) right elbow. **c** Graph depicting progressive deterioration in domains of gross motor function based on the percentage score achieved in the Gross Motor Function Measure (GMFM) and modified Barthel Index. The dashed vertical line indicates the time of initiating oral medication for dystonia and tremor. **d** Brain MRI performed at 13 years of age showing nonspecific T2 hyper-intensities at bilateral parietal deep white matter

The proposita was born at full term with a birth weight of 2900 g and without any perinatal events. Generalized ichthyosis over the torso and extremities was present from birth (Fig. 1a–b), and it was undervalued as being inherited from the father and paternal grandmother, who also had enduring ichthyosis. Although the child experienced febrile convulsion at 10 months, electroencephalography (EEG) at 16 months was normal. A few events of generalized seizures subsided spontaneously, without requiring antiepileptic medications. Brain magnetic resonance imaging (MRI) performed at 2 years of age was normal; however, follow-up MRI at 8 years showed T2-weighted high signal intensities at bilateral parietal deep white matter with mild volume reduction.

Various therapies were applied from the age of 2 years for global developmental delay. Motor delay was remarkable in that she was able to creep by the age of 3 years. Oral anti-spastic medication was started at 4 years of age because of spastic diplegia and was continued for 10 months. At 5 years of age, botulinum toxin was injected twice into her lower limbs which did not show lasting effects. Nonetheless, she was able to walk indoors with ankle-foot orthoses and a walker at 6 years of age. Serial muscle lengthening surgeries on both lower limbs were performed when she was six, 10, and 11 years old; the outcomes were short-term ambulatory improvements only, and contractures remained.

She wore glasses for myopia and photophobia; however, an ophthalmologic evaluation revealed no retinal abnormalities.

Language assessment at 6.3 years of age showed delayed development of receptive language skills compatible with an age of 3.4 years and expressive skills similar to those at an age of 2.4 years. She was able to write her name by the time she was 8 years old. Her intellectual quotient using the Wechsler Intelligence Scale for Children assessed at 13 years of age was a score of 31. At 12 years of age, her independence in activities of daily living scored 57 on the modified Barthel Index.

### Neurodegenerative features

Functional deterioration began when she was 12 years old with the appearance of dystonia and tremor, which were exacerbated with head rotation and forearm pronation, and decline of gait ability. At 15 years of age, with aggravation of dystonia and tremor in the face and upper limbs, dysarthria and upper limb dysfunction became remarkable. Thus, oral medications, pramipexole and baclofen were started; however, only her dystonia was alleviated. Neurological deterioration became apparent when she was 19 years old (Additional file 1: Video S1). A video fluoroscopic swallow test, which was previously normal at 13 years of age, revealed a definite oral and pharyngeal phase delay that required diet modification. Due to aggravated dysarthria, she could only produce vowel sounds. Furthermore, she could not express the desire to defecate or urinate. As a result, her dependence on others for daily living increased, and her modified Barthel Index score was 19 (Fig. 1c).


**Additional file 1:** Video S1. Dystonia and tremor aggravated by age. The video shows dystonic features of the patient at ages 13 and 19 years. As she grew older, the dystonia and tremor aggravated and precipitated the neurological deterioration. (AVI 983 kb)


### Brain imaging studies

To assess the unexplained dystonia and tremor, brain MRI was performed again at 13 years of age, and the results were similar to the previous exam at 8 years old. The last brain MRI for dysphagia at the age of 19 also did not show any change (Fig. 1d). Her ^18^F-fluorodeoxyglucose positron emission tomography scan at 13 years old showed low glucose metabolism in basal ganglia and thalami. In contrast, no abnormal findings were detected by brain MRI in basal ganglia and thalami.

### Electrodiagnostic studies

At 13 years of age, her motor and sensory evoked potentials were all within normal ranges. However, the follow-up study when she was 19 years old revealed delayed motor evoked potentials in all extremities and delayed somatosensory evoked potentials in right side extremities.

Although the initial EEG conducted at 16 months was normal, on serial follow-up, abnormal EEG findings appeared to propagate from intermittent high-amplitude slow discharges in the posterior cerebral region at 8 years of age to sharp discharges from the left parieto-occipital area at 13 years old and then to diffuse cerebral dysfunction compatible with a partial seizure wave at 19 years old. Clinically, she did not have any seizure events after 13 years of age and never required antiepileptic drugs.

### Genetic analyses

**Fig. 2:**
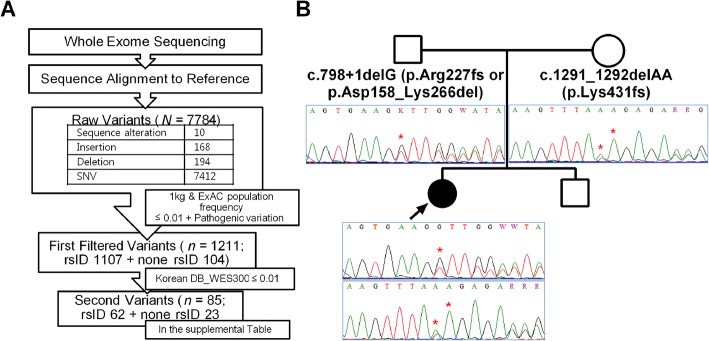
**a** Process of identifying pathogenic genetic variants based on the results of whole-exome sequencing using a population database. **b** Sequence analysis of the *ALDH3A2* gene of the proposita and her parents identified a splicing mutation, 798 + 1delG, from the father and a deletion mutation, 1291-1292delAA, of exon 9 from the mother. One kilogram, 1000 Genomes project (http://www.1000genomes.org/); ExAC, Exome aggregation consortium population databases (http://exac.broadinstitute.org/); rsID, reference single nucleotide polymorphism cluster ID

Whole-exome sequencing was conducted in an attempt to find the cause of neurodegeneration. As a result, 85 variants were identified, including 23 that were not previously reported (Fig. 2a, Additional file 2: Table S1). Two of the variants were in the *ALDH3A2* gene: a c.1291-1292delAA deletion mutation in exon 9 from the mother and a 798 + 1delG splicing mutation from the father, where deletion of the nucleic acid G hinders splicing of intron 5 and results in premature termination of the protein (Fig. 2b). Each mutation was previously reported in homozygous Japanese patients with typical SLS [4]. The other identified genetic variants do not appear to be directly related to neurological deterioration in this case.

## Discussion and conclusions

SLS is a rare hereditary neurocutaneous syndrome with a reported prevalence of 0.4 per 100,000 [4]. Mutation in the *ALDH3A2* gene encoding fatty aldehyde dehydrogenase (FALDH), a microsomal nicotinamide-adenine-dinucleotide-dependent enzyme, is the pathognomic basis for SLS [1]. As oxidation of long-chain aliphatic aldehydes to fatty acids requires FALDH, the symptoms of FALDH-deficient SLS patients are caused by defective clearance of aldehydes [5]. Neurological disturbances may stem from the accumulation of defective eicosanoid metabolite lipids and aldehyde Schiff base in the brain [6]; reversal of reactive aldehydes produced by oxidative stress-induced lipid peroxidation is also defective without FALDH in SLS [7]. One reactive aldehyde, 4-hydroxynonenal, accumulates in the brain tissues of patients with neurodegenerative diseases such as Alzheimer’s disease, Parkinson’s disease, and amyotrophic lateral sclerosis [8]. Accumulated metabolites may have induced neurodegeneration in the present patient, though the exact threshold of reactive aldehyde clearance due to restricted FALDH activity remains unknown.

The proposita in this study exhibited the common features of SLS, such as spastic diplegia and ichthyosis. However, a few manifestations were distinct for the patient, such as neurodegeneration and the occurrence of dystonia with tremor in her early teenage years. Twelve years old, when the patient started to deteriorate, is known as the age of the developmental ceiling for children with SLS [2]. Most patients with SLS are wheelchair-bound due to contractures; however, neuroregression has not been demonstrated with increasing age [9, 10], and the functional level of patients is usually maintained after the developmental plateau [2].

The cause of neurodegeneration and movement abnormalities in our patient was explored with respect to the pathogenesis of SLS and other possible genetic defects through a literature review.

Neurodegeneration has been reported in few children with SLS who required sustained use of anticonvulsants [11] and/or had a history of seizures prior to regression [12]. In those cases, regression occurred at ages of 4 days, 9 months, 4.5 years [11], and 6.5 years [12], much earlier than in our patient. Although apparent seizures were not severe in our patient, neurological deterioration may have been secondary to subclinical seizure activity, as demonstrated with EEG.

Teenage-onset of dystonia with tremor, such as in our proposita, has not been reported previously for SLS. Generalized dystonia was once reported in a Caucasian boy who was diagnosed with SLS at the age of 8 years [13]. The boy’s dystonia was noticeable by the time he was 3 years old, much earlier than in our patient. The boy had two mutations: exon 9 deletion (c.1297-1298 delGA) and exon 6 missense mutation (c.835T > A). Both mutations are known to result in less than 1% residual FALDH activity [14]. The exon 9 deletion in our patient (c.1291-1292 delAA) is in close proximity to the locus in the boy referred to above, with the same 9-bp palindromic sequence [4]. Considering the high prevalence of c.1297-1298 delGA in European SLS families [1], it appears that deletions in exon 9 are less likely to have caused dystonia, despite reduced enzyme activity. Another variant, splicing mutation 798 + 1delG, is also a common mutation reported without dystonia or neurodegeneration. The specific combination of genetic variants in the present patient has not been reported previously and may have resulted in the exceptional clinical course; nonetheless, it is difficult to define a causal relationship. It is known that phenotypic variation is not necessarily related to the type or severity of mutations in SLS patients [15]. A few mechanisms of dystonia may be suggested. White matter disruptions can evoke dystonia due to ephaptic transmission in SLS [13]; disturbed activities, represented as low glucose metabolism, in basal ganglia and thalami may also induce dystonia [16]. Overall, secondary dystonia is a widely noted feature in neurodegenerative conditions and metabolic diseases.

Although the diagnosis of SLS did not fully explain the neurodegeneration in the patient, the results provided better insight to the family. Indeed, the family trait of ichthyosis may have been induced by another ichthyosis-associated gene, as heterozygous carriers of *ALDH3A2* mutations are not symptomatic. This apparently coincidental finding nevertheless is worth mentioning because it hindered early diagnosis. Based on the results of genetic analyses, genetic counseling for a younger brother with a normal phenotype was recommended. To our knowledge, this study is the first to report serial electrophysiological changes in correlation with neurologic deterioration in an SLS patient. A lack of longitudinal follow-up reports on SLS patients due to its rarity may have caused disregard of neurodegeneration as a possible prognostic outcome. Further study to identify the pathophysiology of neurodegeneration in SLS using animal models may provide clues for such clinical manifestations.

## Additional files

Additional file 2: **Table S1.** Pathogenic variations identified in the proposita through whole-exome sequencing. The table reveals all identified variants in our patient including 23 not previous reported. (DOCX 16 kb)

## Abbreviations

EEG: Electroencephalography
FALDH: Fatty aldehyde dehydrogenase
MRI: Magnetic resonance imaging
SLS: Sjogren-Larsson syndrome
