# Supplementary material for: Neurodegeneration in an adolescent with Sjogren-Larsson syndrome: a decade-long follow-up case report
Source: BMC Med Genet. 2018 Aug 29;19:152. doi: 10.1186/s12881-018-0663-0 (PMC6114270; doi:10.1186/s12881-018-0663-0)
Supplement: Supplementary file 2 — Table S1. Pathogenic variations identified in the proposita through whole-exome sequencing. The table reveals all identified variants in our patient including 23 not previous reported. (DOCX 16 kb) [file 12881_2018_663_MOESM2_ESM.docx]

|  | **UnKnown variant (N=23)** | | **Known variant (N=62)** | |
| --- | --- | --- | --- | --- |
|  | **No.** | **Genes** | **No.** | **Genes** |
| **Frameshift variant** | 1 | GDF5 | 3 | ESPN, ALDH3A2, ALDH3A2 |
| **Deletion variant** | 1 | B3GNT3 | 1 | AR |
| **Insertion variant** | 2 | ATN1, GDF5 | 4 | PPP2R2B, CENPP/ASPN, FANCI, MLC1 |
| **Missense variant** | 15 | COL3A1, PRDM9, CNOT4,  CNGB3, TAS2R46, AS2R46,  TAS2R46, TAS2R46, KMT2D,  SBNO1, LOXL1, IQGAP1,  KRT37, NRIP1, EP300 | 41 | PLA2G2D, MUTYH, ADCY10, ZBTB41,  CACNA1S, USH2A, NBAS, LTBP1, NEB,  MLPH, FLNB, KIAA0232, GPR78, CPZ,  GPR98, DST, ROS1, UTRN, ASAH1, TJP2,  TNC, ASS1, RABL6, PTCHD3, SIPA1,  DDX11, KMT2D, AMHR2, SPTBN5,  AKAP13, MYH11, CDT1, GALNS, KRT38,  KLHL10, ITGB4, RNF213, FN3K, ANGPTL4,  CD22, ADARB1, SUN2 |
| **splice_region variant** | 3 | DUOXA2, UBA1, LYST | 12 | DNAJC6, ABP2, GRM1, DIP2C, TPP1,  LDHB, IFNG, TRPC4, PDGFB, ZDHHC15,  ACE, DNM2 |
| **stop_gained variant** | 1 | GDF5 | 1 | TLR4 |

**Table S1 Pathogenic variations identified in the proposita through whole-exome sequencing**
